# Fabrication of a Triple-Layer Bionic Vascular Scaffold via Hybrid Electrospinning

**DOI:** 10.3390/jfb15060140

**Published:** 2024-05-23

**Authors:** Feier Ma, Xiaojing Huang, Yan Wang

**Affiliations:** 1 School of Sports Medicine and Rehabilitation, Beijing Sport University, Beijing 100084, China; 2 Institute of Orthopaedic & Musculoskeletal Science, Division of Surgery and Interventional Science, University College London, The Royal National Orthopaedic Hospital, Stanmore, London HA7 4LP, UK

**Keywords:** bionic scaffold, wet electrospinning, vascular scaffold, fiber scaffold, tubular scaffold, small-diameter scaffold

## Abstract

Tissue engineering aims to develop bionic scaffolds as alternatives to autologous vascular grafts due to their limited availability. This study introduces a novel wet-electrospinning fabrication technique to create small-diameter, uniformly aligned tubular scaffolds. By combining this innovative method with conventional electrospinning, a bionic tri-layer scaffold that mimics the zonal structure of vascular tissues is produced. The inner and outer layers consist of PCL/Gelatin and PCL/PLGA fibers, respectively, while the middle layer is crafted using PCL through Wet Vertical Magnetic Rod Electrospinning (WVMRE). The scaffold’s morphology is analyzed using Scanning Electron Microscopy (SEM) to confirm its bionic structure. The mechanical properties, degradation profile, wettability, and biocompatibility of the scaffold are also characterized. To enhance hemocompatibility, the scaffold is crosslinked with heparin. The results demonstrate sufficient mechanical properties, good wettability of the inner layer, proper degradability of the inner and middle layers, and overall good biocompatibility. In conclusion, this study successfully develops a small-diameter tri-layer tubular scaffold that meets the required specifications.

## 1. Introduction

Cardiovascular disease is responsible for the deaths of 17 million people annually, with arteriosclerosis being one of the primary causes of this disease [1]. Medication and surgery are the current treatment options for patients suffering from symptomatic arterial thrombosis [2,3]. However, the availability of autologous vessels is limited or inadequate due to the patient’s health status or previous surgical history of vascular grafting [4,5]. Non-degradable artificial vascular implants are commonly used in surgical interventions [6,7]. Nevertheless, tissue inflammation and fibrosis can lead to reduced implant survival rates in patients over a 10-year period [8]. To overcome the limitations associated with the use of non-degradable implants and the scarcity of alternative vascular implants [9,10], tissue engineering has developed degradable vascular scaffolds that can replace autologous vessels [6]. While large-diameter synthetic vascular scaffolds have shown promising clinical outcomes [11], the clinical application of small-diameter (<6 mm) scaffolds still poses significant challenges [12].

The electrospinning technique is widely employed to produce fiber scaffolds that mimic the extracellular matrix (ECM). These bioengineered scaffolds, which closely resemble the morphology of natural tissues, are conducive to cell proliferation [13]. However, the fabrication of three-dimensional biomimetic structures using this technique remains an ongoing challenge [14]. For blood vessels, they are zonal structures. The arterial wall is composed of three layers, namely the tunica intima, tunica media, and tunica adventitia [15]. The intima directly interacts with blood and functions as an anti-thrombotic barrier [16], characterized by the random arrangement of ECM components [17]. The tunica media consists of smooth muscle cells (SMCs) and exhibits a parallel arrangement of the ECM [18]. The outer wall of blood vessels is composed of fibroblasts, exhibiting a random arrangement of the ECM [19]. In this trial, the fiber arrangement and functionality of each bioscaffold layer are designed and prepared based on the characteristics of vascular tissues. 

The traditional electrospinning equipment, employing a high-speed rotating rod to collect aligned fibers, faces challenges in controlling fiber alignment due to the long distance between the electrodes [20,21]. The formation of fibers is influenced by both electrical and non-electrical parameters [22]. Compared to the fabrication of flat fiber mats, the fabrication of small-diameter tubular scaffolds requires a higher rotation speed [23], which often results in an uneven distribution of fibers, with thinner outer areas compared to the middle [24]. In contrast, the wet electrospinning technique allows for the physical rearrangement of fiber alignment. This approach stabilizes fiber collection by increasing fluid volume and utilizes a liquid bath collector to accelerate fiber coagulation [25], resulting in a specific fiber arrangement [26]. We propose that this method can yield highly porous fibrous scaffolds in a stable manner by controlling the flow rate of the collecting fluid. Furthermore, this technique is efficient and cost-effective for spinning and aligning fibers on the collector, leading to an orderly and parallel fiber arrangement.

This experiment aims to fabricate a small-diameter three-layer vascular scaffold that mimics the natural vessels. The innovative fabrication method and the design of the material used to fabricate the triple-layer bionic scaffold can be structurally matched to natural vascular tissue. PCL offers high mechanical properties and biocompatibility but lacks cell recognition sites and exhibits gradual degradation in vivo [27]. To address these constraints, we blended PCL with gelatin and heparin. Gelatin provides high biodegradability and biocompatibility [28,29], while heparin binds growth factors electrostatically, promoting local angiogenesis and endothelial cell proliferation [30]. For the outer vascular layer’s mechanical requirements, PCL was combined with PLGA.

An innovative method, which combined the electrospinning technique and Wet Vertical Magnetic Rod Electrospinning (WVMRE) to fabricate different layers, was used. By combining conventional electrospinning with this innovative method, a bionic tri-layer scaffold was created to mimic the zonal extracellular matrix of vascular tissues. A roller collector in this technique is driven by magnetic stirring to collect well-aligned PCL fibers to simulate the vascular middle layer, while the disordered structure of the inner and outer layers is fabricated by a rotating-rod electrospinning technique with PCL/gelatin/heparin and PCL/PLGA. Eventually, the bionic triple-layer vascular scaffold matched the natural vascular ECM structure and mechanical characteristics. In addition, this scaffold had good wettability and biocompatibility to support cell proliferation. Each fiber layer of this bionic scaffold had a certain degradation capacity, reducing the risk of tissue inflammation [31].

## 2. Materials and Methods

### 2.1. Materials 

N,N-Dimethylformamide (DMF), chloroform, polycaprolactone (PCL), gelatin, poly(lactic-co-glycolic acid) (PLGA), acetic acid, hydrochloric acid, Hexafluoro-2-propanol (HFIP), and Endothelial Cell Growth Medium were purchased from Sigma company (Darmstadt, Germany). N-Hydroxy succinimide (NHS), 1-Ethyl-3-(3-dimethylaminopropyl)-carbodiimide (EDC), 2-Morpholinoethanesulphonic acid (MES), phosphate-buffered saline (PBS), LIVE/DEAD^®^ Viability/Cytotoxicity Kit and PrestoBlue^TM^ Cell Viability Reagent were purchased from Thermo Fisher (Waltham, MA, USA). The cell culture consumables were purchased from PromoCell (Heidelberg, Germany). The human umbilical vein endothelial (HUVE) cells were obtained from University College London (London, UK). 

### 2.2. Set-Up

#### 2.2.1. Wet Vertical Magnetic Rod Electrospinning (WVMRE)

**Figure 1:**
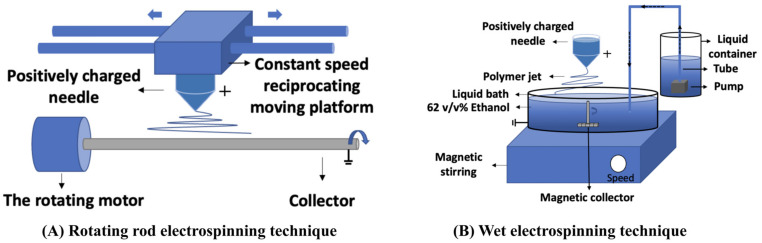
The fabrication set-up. (**A**) The rotating-rod electrospinning technology: The needle is synchronized with the platform to drive the spinning fibers across the collector. The collector, rotating with the assistance of a motor, collects the fibers in a random arrangement. (The blue arrow shows the direction of the device’s movement.) (**B**) Wet Vertical Magnetic Rod Electrospinning (WVMRE): The magnetic collector, driven by a magnetic stirrer, rotates to collect the fibers that float on the alcohol surface. The fibers are collected in a parallel arrangement on the collector. (The blue arrow shows the direction of the device’s movement).

The novel apparatus presented in Figure 1B is engineered to fabricate highly aligned tubular fibers, as depicted in Figure 1B. The innovative collector is composed of three components: a grounded liquid bath, a liquid reservoir equipped with an internal pump containing a 62% ethanol solution, and a vertically mounted rod (made from polytetrafluoroethylene with a diameter of 6 mm and a length of 4 cm) driven by a magnet mounted on the bottom of the rod.

When a high voltage was applied to the needle, electrospun fibers were deposited onto the surface of the ethanol. These fibers were wetted and floated on the liquid surface. Concurrently, a magnetic stirrer drove the rod, generating a dynamic fluid flow that propelled the liquid from the edge to the center. This prompted the non-woven fibers to rearrange into an aligned configuration while becoming entangled with the rod at the interfacial region between the rod and the liquid. This interface level was progressively controlled through liquid transfer from the internal pump, resulting in a uniformly thick 3D matrix.

We investigated the impact of different flow rates on the porosity and thickness of the textile scaffold during the production process. The liquid was transferred from the reservoir to the bath using three rates: low, medium, and high. The porosity and thickness of the produced scaffold were measured using calculation formulas and a caliper.

#### 2.2.2. Scaffold Fabrication

**Figure 2:**
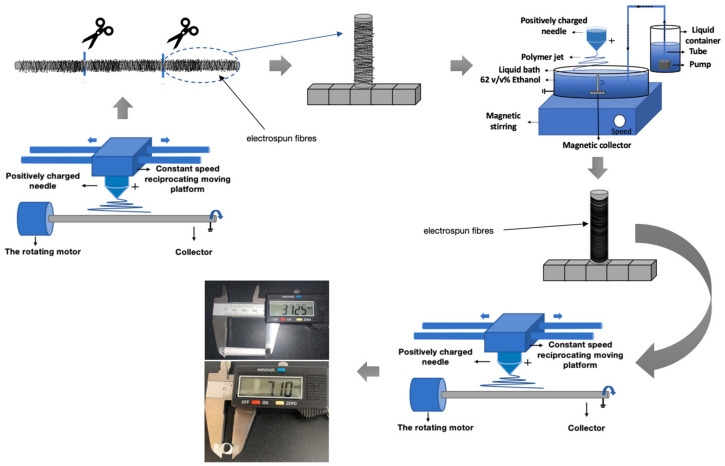
The process of triple-layer scaffold fabrication. (The blue arrow shows the direction of the device’s movement; the grey arrows show the sequence of the experimental process).

A three-layer vascular scaffold was fabricated using the conventional rotating-rod electrospinning technique and WVMRE (Figure 2, Table 1). 

In this trial, three different materials were used: PCL (mw = 8000, Sigma), gelatin, and PLGA to fabricate the three-layer scaffold. PCL and gelatin were used to fabricate the inner layer of the biomimetic scaffold. For the HFIP solvent system, 24 wt% PCL and gelatin (1:1) were dissolved separately. Then, PCL and gelatin solutions were mixed after 24 h stirring, and 0.2 *v*/*v*% acetic acid was added to the co-mixing solution to keep a homogeneous mixture [30]. The inner layer was fabricated via the roller electrospinning technique. The solutions were electrospun at 0.4 mL/h and 13 kV. The inner-layer fibers (PCL and gelatin, PG) were collected on a rotating rod with a diameter of 6 cm. 

To improve the biocompatibility of the fabricated scaffold, heparin was crosslinked to the inner layer. The 0.1 M MES buffer was first prepared in advance with pH = 7. Depending on the sample weight, NHS and EDC (NHS:EDC = 2.5:1, EDC:heparin = 1:1) were added to the MES buffer and cooled in the refrigerator at 4. The heparin was weighted according to the sample mass (heparin:gelatin = 1:1), and the heparin was dissolved in the prepared activation solution. The samples were immersed in the prepared solution overnight. After overnight, the samples were washed 3 times with PBS and then evacuated. Successful crosslinking of heparin on the scaffold was demonstrated in the TBO experiments. The color of the sample with heparin was different than the color of the sample without heparin after the TBO experiment. The absorbance of the TBO supernatant on the samples was significantly different. 

For the middle layer, PCL was used to fabricate via WVMRE; 13 wt% PCL was dissolved in DMF/chloroform (2/8 *v*/*v*). This solution was electrospun at 1.5 mL/h and 13 kV. Then, the fibers were collected in a liquid bath of 62% alcohol (Figure 1B). The second-layer fibers were arranged circumferentially on the first layer of the scaffold.

For the outer layer (PCL-PLGA), PCL and PLGA were used to fabricate via the rotating-rod electrospinning technique (Figure 1A, Table 1). First, 26 wt% PCL and PLGA (75:25) were dissolved in DMF/chloroform (2/8 *v*/*v*). The solution was then electrospun at 0.4 mL/h and 13 kV. The outer layer was arranged randomly on the middle layer of the scaffold. The triple-layer bionic scaffold was fabricated.

#### 2.2.3. Morphology

The gravity method was used to measure the porosity, as per the following equation [32]:

(1) $$\mathrm{The}\;\mathrm{density}\;\mathrm{of}\;\mathrm{scaffold}\;(g/{\mathrm{cm}}^{3})=\frac{\mathrm{Mass}\;\mathrm{of}\;\mathrm{scaffold}\;(g)}{\mathrm{Surface}\;\mathrm{area}\;\mathrm{of}\;\mathrm{the}\;\mathrm{sample}\;({cm}^{2})\times \;\mathrm{Thickness}\;\mathrm{of}\;\mathrm{scaffold}\;(\mathrm{cm})}$$

(2) $$\frac{1}{\rho_{\mathrm{Polymers}\;\mathrm{used}}}=\frac{{wt\%}_{PCL}}{\rho_{PCL}}+\frac{{wt\%}_{Additional\;material}}{\rho_{Additional\;material}}$$

(3) $$\mathrm{Porosity}\;\mathrm{of}\;\mathrm{scaffold}\;=1-\frac{\mathrm{The}\;\mathrm{density}\;\mathrm{of}\;\mathrm{scaffold}\;(g/{cm}^{3})}{\mathrm{The}\;\mathrm{density}\;\mathrm{of}\;\mathrm{polymers}\;\mathrm{used}\;(g/{cm}^{3})}\;$$

where the additional material is PCL (ρ=1.021), gelatin (ρ=1.34) [33] in inner layer, and PLGA (ρ=1.25) [34] in outer layer.

#### 2.2.4. FTIR

PCL, PLGA, gelatin, and fibers of three layers were characterized via Fourier-transformed infrared (FITR) spectra, and samples were measured at wavelengths of 4000–450 cm^−1^ with a resolution of 4 cm^−1^.

#### 2.2.5. Water Contact Angle

The contact angle of PG, PG with Heparin (PGH), PCL, and PCL-PLGA fiber mats was measured by light microscopy (axioskop 2 Plus, Zeiss, UK): 5 μL of deionized water was dropped on the surface of fiber mats, and the whole process was recorded under the microscope. The contact angle was calculated by ImageJ software (version 1.8.0). 

#### 2.2.6. Tensile Test

The mechanical characterization of samples was carried out using a universal tensile test machine (Zwick Roell, Ulm, Germany) with a crosshead speed of 50 mm/min to measure the Young’s modulus, elongation at break (%), and maximum tensile stress (MPa). After the sample was cut into 1 × 1.5 cm^2^, the tests were performed in the axial and radial directions. The cross-sections and longitudinal sections of samples were measured.

#### 2.2.7. Material Degradation

The weight loss for each layer and a degradation test for gelatin in the inner layer were conducted. Samples from each layer were immersed in PBS, and weight loss was measured after 12 h, 1 day, 3 days, 7 days, and 14 days of immersion. The percentage of weight loss was calculated using the following equation:

(4) $$\mathrm{The}\;\mathrm{percentage}\;\mathrm{of}\;\mathrm{weight}\;\mathrm{loss}=\frac{\left(w_{t}-w_{0}\right)}{w_{o}}\times 100\%$$

where $w_{t}$ and $w_{0}$ were the original weight of samples and the weight of degraded samples, respectively.

The inner-layer samples were tested for gelatin content after the weight loss test. The hydroxyline concentration test was used to confirm the remaining gelatin concentration. The samples were hydrolyzed with 6 M hydrochloric acid for 48 h at 110 °C. The samples were heated in a water bath for 20 min after sequentially adding diluent, oxidizing agent, and color reagent. After a 10 min incubation period, the absorbance of the samples was measured using a UV-Vis spectrophotometer (Shimadzu, MK, UK) in the wavelength range of 400–4000 cm^−1^. The gelatin contents of the degraded scaffold were determined according to an optimized gelatin content-absorbance standard curve.

### 2.3. In Vitro Evaluation

#### 2.3.1. Cell Culture and Seeding

The human umbilical vein endothelial (HUVE) cell culture medium is composed of endothelial cell growth basal medium, SupplementMix, 10 *v*/*v*% Fetal Bovine Serum, and 1 *v*/*v*% antibiotic. The P5~10 HUVE cells were cultured on a 125 cm^2^ flask in culture medium and placed in a 37 °C, 5% CO_2_ incubator. The culture medium was changed once every three days. Subsequently, HUVE cells were seeded at a density of 10,000 cells per milliliter on scaffolds, both with and without crosslinked heparin. After a 4 h incubation in a CO_2_ incubator to facilitate cell adhesion, cell culture was initiated by adding 400 μL of culture medium to each well.

#### 2.3.2. Cell Morphology and Viability Tests

A scanning electron microscope (SEM; Phenom ProX, Waltham, MA, USA) was used to observe the morphology of the seeded cells at D1 and D7. Subsequently, cell activity was analyzed via the LIVE/DEAD Viability/Cytotoxicity Kit. EthD-1 was used to stain dead cells, and calcerin AM was used to stain live cells. A fluorescent microscope (axioskop 2 Plus, Zeiss, Birmingham, UK) was used to demonstrate cell viability. Cell proliferation was demonstrated by comparing the activity of cells inoculated on the scaffold surface on Day 1 and Day 7.

### 2.4. Statistical Analysis

The results of each test are presented as mean ± SD. Graph Prism 9^®^ was used for data analysis and plotting. The unpaired *t*-test and one-way ANOVA were used to determine significant differences (*p* value). The results were determined to be significantly different when *p* < 0.05. Image J (version 1.8.0) was used for image analysis. The scale bar in the image is 200 µm in length. 

## 3. Results

### 3.1. Technical Optimization

**Figure 3:**
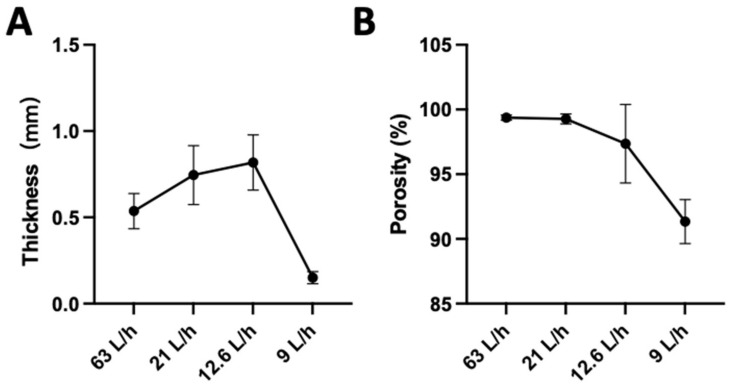
The characteristics of fiber mats at different fabrication times. (**A**) The PCL porosity at four fabrication times. (**B**) The PCL thickness at four fabrication times.

The porosity of PCL fibers fabricated at four durations is shown in Figure 3. The results show that fabrication duration can significantly affect sample porosity and thickness (*p* < 0.05). There is a declining tendency in sample porosity and thickness with increasing fabrication duration. The PCL fibers produced using this technology are high-porosity materials.

### 3.2. Characterization of Scaffold

#### 3.2.1. Morphology

**Figure 4:**
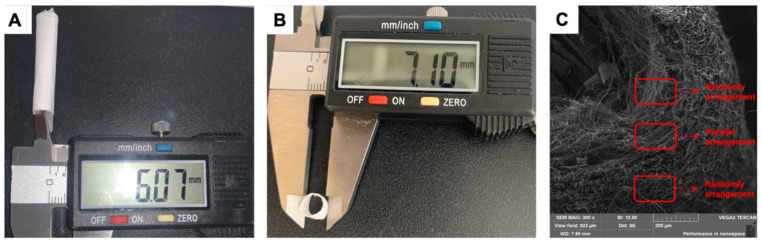
The characterization of the scaffold. (**A**) The inner diameter of the spinning scaffold. (**B**) The outer diameter of the spinning scaffold. (**C**) The fiber arrangement image of the spinning mat.

This vascular scaffold is a thin, tubular, hollow structure, and its internal diameter is 6 mm (Figure 4A,B). Each layer’s fiber structure mimics the ECM of each vascular intima (Figure 4C). The internal and external fibers are randomly aligned; the middle fibers are arranged in parallel. This scaffold has high porosity and low pore size to support cell adhesion and proliferation. The results of the characterization of the scaffold and each layer are shown in Table 2.

#### 3.2.2. Scaffold Composition

**Figure 5:**
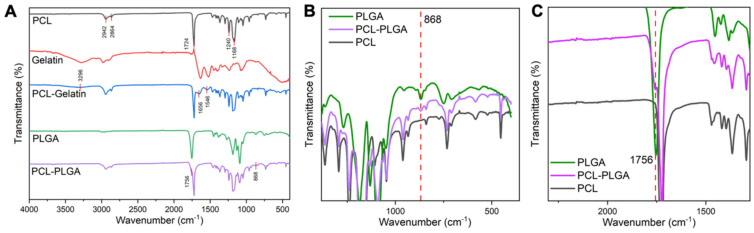
(**A**) The FTIR spectra for each layer and material. (**B**) The characteristic peak of PLGA at 868 cm^−1^. (**C**) The characteristic peak of PLGA at 1756 cm^−1^.

The FTIR spectra for the three layers of fibers of this scaffold are shown in Figure 5A. This three-layer scaffold was fabricated with PCL as its main component. 

The first line is the middle PCL, which shows characteristic peaks of PCL at 2942 cm^−1^ (for CH2 asymmetric stretching vibrations), 2864 cm^−1^ (associated with CH_2_ symmetric stretching vibrations), 1724 cm^−1^ (for C=O stretching), 1240 cm^−1^ (for C-O-C asymmetric stretching), and 1168 cm^−1^ (for C-O-C symmetric stretching) [35]. In the inner PCL-Gelatin fiber, all characteristic bands of PCL and gelatin were observed. For example, 3296 cm^−1^ (for N-H stretching in amide A), 1656 cm^−1^ (for C=O stretching in amide I), and 1546 cm^−1^ (for N-H in-plane bending in amide II) [36]. The characteristic bands of PCL and PLGA were observed for the outer layer at the last line. The characteristic bands that could distinguish PCL and PLGA are 865 cm^−1^ (for C-H bends) (Figure 5B) and 1756 cm^−1^ (association with C=O stretching) (Figure 5C) [37,38]. The FTIR spectra of PCL-PLGA electrospun fibers overlapped with all peaks of PCL and PLGA, indicating the successful mixing of PCL and PLGA in this fiber layer. These data show that three layers of electrospun fibers were successfully manufactured in this experiment, and the material in each layer was consistent with the experimental design.

#### 3.2.3. Water Contact Angle

**Figure 6:**
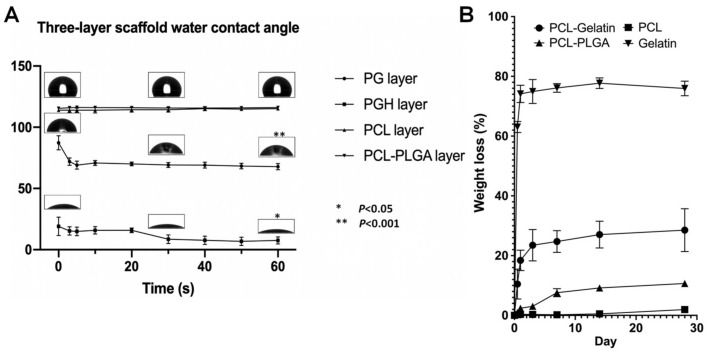
(**A**) The water retention capability of each layer. (**B**)The degradation curve of materials.

Figure 6A demonstrates the water contact angle for each layer of this three-layer scaffold and the wettability of each layer. The WCA of the PCL fiber mat was 113.88 ± 1.30°, which shows hydrophobicity immediately after water contact with the fiber mat and 115.18 ± 1.35° after 60 s. There was no significant change. The WCA of the PCL-PLGA fiber mat was 115.40 ± 0.74° immediately after water contact with the fiber mat and 116.00 ± 1.07° after 60 s. There was no significant change. The WCA of the PCL-gelatin fiber mat was 87.28 ± 5.73°, which shows excellent hydrophilicity immediately after water contact with the mat, and 69.20 ± 1.98° after 60 s, which shows a very significant decrease. The WCA of the PCL-gelatin-heparin fiber mat was 19.04 ± 7.55°, showing better hydrophilicity than the PCL and gelatin fiber mat immediately after water contact with the mat and 7.6 ± 2.89° after 60 s, which is a significant reduction. 

#### 3.2.4. Material Degradation

The weight loss of the material is shown in Figure 6B. The fiber weight loss results show that the inner layer of the scaffold has good biodegradability, and the inner layer is degraded by gelatin. The weight loss of fiber mats occurred primarily in the first week, from 10.48 ± 5.00% to 24.73 ± 3.62%. The comparison of the degradation curves for the weight loss between the inner and middle fibers shows that the main material degraded in the inner fibers is gelatin. The main degradation of gelatin occurred within 12 h, and then it continued to degrade slowly. After 12 h, the gelatin content had significantly decreased. Subsequently, the degradation rate of gelatin slowed, and there was no significant change in gelatin content compared to after 12 h. Therefore, the main material degraded in the inner layer of fibers was gelatin. 

The middle layer was less biodegradable than the three layers in terms of degradability. There was no significant difference in mass loss for the middle layer over 28 days. The outer layer showed improved biodegradability compared to the middle layer. For the outer-layer fibers, the degradation rate of the fibers was faster in the first week, and then the degradation rate became slow and consistent with the PCL degradation curve.

#### 3.2.5. Mechanical Property Test

The mechanical properties of each layer of this scaffold are shown in Table 3. The middle layer of fibers has a significant difference in Young’s modulus between the axial and radial directions of the layer due to its manufacturing process (5.13 ± 10.02 MPa and 62.09 ± 550.40 MPa, respectively; *p* < 0.001). The fabrication techniques of the remaining two layers do not significantly affect the mechanical properties in the axial and radial directions. There was no significant difference in the Young’s modulus between the axial and radial directions for the three-layer scaffold (*p* > 0.05). There was also no significant difference between the axial and radial elongations at break for the three-layer stent (*p* > 0.05), and this property met the requirements of the vascular tissue (elongation at break over 40%) [39]. The mechanical properties of the scaffold showed that the scaffold could meet the mechanical requirements of scaffolds in blood vessels and that the mechanical properties of the scaffold layers matched the mechanical properties of the three vascular layers. 

### 3.3. In Vitro Evaluation

#### Cell Morphology and Viability Tests

**Figure 7:**
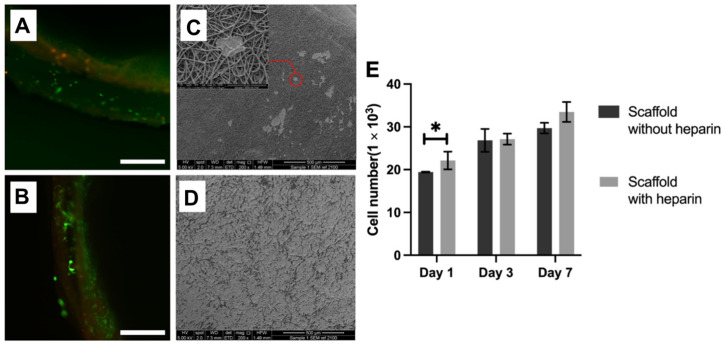
The live and dead images of cells and the proliferation results. (**A**) Day 1 cells, dead and live images. (**B**) Day 7 cells, dead and live images. (**C**) Day 1 HUVE cell adhesion on crosslinked heparin scaffold. (**D**) Day 7 HUVE cell adhesion on crosslinked heparin scaffold. (**E**) Comparative analysis of HUVE cells on Day 1, 3, and 7, with and without crosslinked heparin material, * *p* < 0.05.

The cytofluorograms of the cells cultured on the scaffold surface on the first and seventh days show the good cellular compatibility of this scaffold (Figure 7). Figure 4A shows that the majority of the cells grown on the first day were live cells, and most of the cells adhered to the fiber surface. The number of cells on the seventh day increased compared to the first day (Figure 7E). This result indicates that the scaffold has good biocompatibility. The cross-section of the fibers shows that HUVE cells successfully adhered to the inner layer of the fibers.

This study conducted SEM examinations to investigate the morphology and expansion of HUVE cells on the scaffold surface on the first day of the inoculated HUVE cell scaffold (Figure 7). The results show an adequate expansion of HUVE cells on the scaffold (Figure 7D). These results indicate that the scaffold is biocompatible and can support endothelial cell adhesion and proliferation.

## 4. Discussion

Tissue engineers have designed scaffolds to advance cardiovascular treatments [1,2], with large-diameter vascular scaffolds showing promising clinical outcomes [40,41]. However, challenges persist in the clinical application of small-diameter (<6 mm) scaffolds [42]. Thus, it is crucial to develop a small-diameter vascular bionic scaffold to update the surgical treatment options for arteriosclerosis. In this study, a small-diameter triple-layer vascular bionic scaffold was fabricated according to the structure and properties of natural vessels. The fabricated scaffold has good biocompatibility and mimics the mechanical properties and structure of natural vessels, which provides the potential for small-diameter scaffold implantation as a surgical option. 

This work applied an innovative wet electrospinning technique to fabricate a precisely aligned tubular scaffold. Unlike conventional wet-electrospinning approaches, which typically yield planar fiber mats [43], our work presents a modified technique aimed at achieving a specific 3D tubular architecture. Additionally, our innovative wet-electrospinning technique produced aligned fiber arrangements, as opposed to the random arrangement of fibers obtained via conventional wet electrospinning [43]. Wang [26] also utilized a similar wet-electrospinning method to fabricate an aligned fiber scaffold, but they still fabricated planar fiber mats. Notably, our work utilized smaller rotational speeds and equipment compared to Wang’s study, allowing us to achieve the 3D tubular scaffold structure with a parallel arrangement of fibers.

The parallel arrangement of fibers in this work showed weaker mechanical properties due to reduced physical bonding, a finding consistent with Tong [44]. To enhance the middle-layer fiber mechanical properties, bionic composite structures have been employed. Our work mitigated the influence of the middle layer by utilizing multiple fiber layers. As a result, the mechanical properties of the triple-layer scaffold were improved, and the differences in the axial and radial directions did not adversely affect the scaffold. 

A 1 mm thick 3D tubular structure was successfully fabricated using the parameters of 0.4 mL/h and 13 kV in the rotating-rod electrospinning technique and 1.5 mL/h and 13 kV in the WVMRE technique. However, there was a decreasing tendency in sample porosity with increasing fabrication time. This phenomenon may be due to the stretching and bonding of fibers [45]. The stretching process effectively reduces fiber thickness [46], promoting a dense arrangement among them [47] and ultimately leading to lower porosity [48,49]. Moreover, our method ensures a better uniform fiber distribution compared with other rotated electrospinning methods. The fiber deposition only happened at the liquid level. As a result, the feed rate of the liquid controlled the thickness [50]. Also, heparin was added for blood compatibility. Heparin plays a key role in inhibiting the activation of coagulation factors and can effectively alleviate the formation of a thrombus on the surface of the scaffold [51,52]. 

This study’s findings demonstrate the possibility of the clinical application of small-diameter vascular scaffolds and provide a new fabrication technology method for the production of small-diameter vascular scaffolds. Furthermore, the fabrication of the scaffold remains to be improved in some areas. In this work, the three layers of the scaffold were stacked sequentially without establishing proper interlayer bonding. Consequently, the fibers within each layer did not experience simultaneous breakage during the experimental evaluation. In future studies, exploring solutions for enhancing interlayer connections should be a focal point of investigation. 

## 5. Conclusions

To explore innovative surgical options for small-diameter bionic vascular scaffolds, this study successfully developed a three-layer bionic vascular scaffold using a combination of rotating-rod electrospinning and Wet Vertical Magnetic Rod Electrospinning. This approach facilitated the creation of a highly porous fibrous matrix that closely resembles the natural vascular extracellular matrix, fostering the regeneration of vascular cells within the scaffold.

By integrating this novel technique with rotating-rod electrospinning, a 3D hollow tubular scaffold was effectively fabricated that closely mimics the morphology and extracellular matrix of natural vessels. Notably, this scaffold exhibits mechanical properties that maintain vascular function, also demonstrating controlled degradation attributes to mitigate immune responses.

The findings of this study demonstrated the clinical potential of scaffolds produced through this technology. Future investigations should focus on enhancing interlayer cohesion to bolster scaffold stability with subsequent in vivo evaluations.

In conclusion, these findings illuminate a range of potential clinical surgical protocols leveraging the potential applications of this technology. 

## Figures and Tables

**Table 1:** The parameters of electrospinning and wet-electrospinning fabrications.

Layers	Materials	Polymer Solution		Electrospinning Parameters		
		Polymer Solvent	Polymer Concentration (wt%)	Flow Rate (mL/h)	Distance (cm)	Voltage (kV)
Inner layer	PCL/Gelatin	HFIP	24 (PCL:Gelatin = 1:1)	0.4	15	13
Middle layer	PCL	DMF/chloroform (2/8 *v*/*v*)	13	1.5	12	13
Outer layer	PCL-PLGA	DMF/chloroform (2/8 *v*/*v*)	26 (PCL:PLGA = 75:25)	0.4	15	13

Scaffold mechanical characterization.

**Table 2:** The characterization of pore size and porosity and the thickness of each layer (*n* = 6).

	Porosity (%)	The Thickness of Each Layer (μm)
PCL-Gelatin	71.51 ±2.68	49.50 ± 13.66
PCL	78.44 ±1.25	158.67 ± 21.32
PCL-PLGA	76.14 ±1.45	63.50 ± 14.28

**Table 3:** The mechanical properties of the scaffold and each layer of the scaffold.

Layer	Axial Young’s Modulus (MPa)	Radial Young’s Modulus (MPa)	Axial Maximum Tensile Strength (MPa)	Radial Maximum Tensile Strength (MPa)	Axial Elongation at Break (%)	Radial Elongation at Break (%)
PCL- Gelatin-Heparin	26.76 ± 79.36	32.13 ± 143.02	4.08 ± 1.51	6.08 ± 1.96 *	341.61 ± 92.01	383.70 ± 42.94
PCL	5.13 ± 10.02	62.09 ± 550.40 ***	0.4 ± 0.02	4.26 ± 2.85 **	198.29 ± 106.80	156.28 ± 77.00
PCL- PLGA	16.28 ± 5.04	12.35 ± 5.75 *	1.47 ± 0.008	0.95 ± 0.03 ***	185.50 ± 23.70	49.16 ± 7.52 ***
Three- layer	13.89 ± 13.01	10.67 ± 19.35	1.59 ± 0.14	2.97 ± 0.30 **	378.50 ± 122.20	454.20 ± 133.10

* *p* < 0.05; ** *p* < 0.01; *** *p* < 0.001.

## Data Availability

The raw data supporting the conclusions of this article will be made available by the authors on request.
